# Aberrant Expression of Shared Master-Key Genes Contributes to the Immunopathogenesis in Patients with Juvenile Spondyloarthritis

**DOI:** 10.1371/journal.pone.0115416

**Published:** 2014-12-15

**Authors:** Lovro Lamot, Fran Borovecki, Lana Tambic Bukovac, Mandica Vidovic, Marija Perica, Kristina Gotovac, Miroslav Harjacek

**Affiliations:** 1 Department of Pediatrics, University of Zagreb School of Medicine, Zagreb, Croatia; 2 Division of Pediatric and Adolescent Rheumatology, Children's Hospital Srebrnjak, Zagreb, Croatia; 3 Department for Functional Genomics, Center for Translational and Clinical Research, University of Zagreb School of Medicine, Zagreb, Croatia; University of Jaén, Spain

## Abstract

Association of juvenile spondyloarthritis (jSpA) with the HLA-B27 genotype is well established, but there is little knowledge of other genetic factors with a role in the development of the disease. To date, only a few studies have tried to find those associated genes by obtaining expression profiles, but with inconsistent results due to various patient selection criteria and methodology. The aim of the present study was to identify and confirm gene signatures and novel biomarkers in highly homogeneous cohorts of untreated and treated patients diagnosed with jSpA and other forms of juvenile idiopathic arthritis (JIA) according to ILAR criteria. For the purposes of the research, total RNA was isolated from whole blood of 45 children with jSpA and known HLA genotype, 11 children with oligo- and polyarticular forms of JIA, as well as 12 age and sex matched control participants without diagnosis of inflammatory disease. DNA microarray gene expression was performed in 11 patients with jSpA and in four healthy controls, along with bioinformatical analysis of retrieved data. Carefully selected differentially expressed genes where analyzed by qRT-PCR in all participants of the study. Microarray results and bioinformatical analysis revealed 745 differentially expressed genes involved in various inflammatory processes, while qRT-PCR analysis of selected genes confirmed data universality and specificity of expression profiles in jSpA patients. The present study indicates that jSpA could be a polygenic disease with a possible malfunction in antigen recognition and activation of immunological response, migration of inflammatory cells and regulation of the immune system. Among genes involved in these processes TLR4, NLRP3, CXCR4 and PTPN12 showed almost consistent expression in study patients diagnosed with jSpA. Those genes and their products could therefore potentially be used as novel biomarkers, possibly predictive of disease prognosis and response to therapy, or even as a target for new therapeutic approaches.

## Introduction

Spondyloarthritis (SpA) is a term that comprises a group of seronegative, immune-mediated inflammatory disorders with similar clinical and genetic manifestations [1]. These diseases are characterized by enthesitis and arthritis affecting the joints of the lower extremities and seronegativity for IgM rheumatoid factor and antinuclear antibodies. The SpA family of diseases includes ankylosing spondylitis (AS), reactive arthritis (ReA), psoriatic arthritis (PsA), arthritis associated with inflammatory bowel disease (IBD), undifferentiated SpA and a juvenile form of SpA (jSpA). The latter, according to ILAR (The International League of Associations for Rheumatology) classification of juvenile idiopathic arthritis (JIA), are classified as enthesitis-related arthritis (ErA), psoriatic arthritis (PsA) or undifferentiated arthritis [2]. Nevertheless, the majority of jSpA patients can be classified as ErA, which often leads to interchangeable use of terms. SpA often begins as “undifferentiated” with different manifestation in children and adults; most notably, spinal involvement is uncommon, while hip arthritis is frequently seen in the juvenile-onset disease [3]. As a consequence, jSpA might be missed or confused with other forms of juvenile arthritis.

SpA is a multifactorial disease in which a disturbed interplay occurs between the immune system and environmental factors on a predisposing genetic background, which is dominated by one family of MHC class I alleles, HLA-B27. This genotype accounts for close to 40% of heritability in AS and seems almost necessary for the disease development (present in more than 90% of AS patient compared with 7–8% healthy controls), but is clearly not sufficient, since only 5% of HLA-B27 carriers develop AS [4]. A similar role has also been proposed for HLA-B7 [5], [6]. In our previous cohort study of 74 children from Croatia diagnosed with jSpA/ErA, odds ratio (OR) for disease development were calculated according to presence of HLA-B27 allele, HLA-B7 allele, or both [7]. It has been shown that children with HLA-B7 allele have 2.61 times higher odds for disease development than children from general Croatian population without this allele. The odds were 5.69 times higher for children with HLA-B27 allele, and even 14.82 times higher if child had both HLA-B7 and HLA-B27 alleles.

Majority of studies today use the high-throughput methods that allow us to study genes on a global scale. Recently, one of these methods was used to perform a case-control association study in adult patients with AS [8]. The results confirmed some genes previously identified in patients with SpA, such are ERAP1, IL23R, IL12B, STAT3, PTGER4 and CARD9 [9]. These studies assessed the association between a common single nucleotide polymorphism (SNP) and a complex disease. However, to understand the disease mechanism and close the gap between genotype and phenotype, other genomic data, such as quantification of gene expression, is often necessary. Gene expression profiling generates a “snapshot” of cellular activity at the time of analysis, telling us exactly what processes are occurring. By comparing disease and control samples it is possible to elucidate the processes contributing to the disease and how they are altered [10]. There are several methods of gene expression profiling, among which DNA microarray is still the most common, accounting for almost 75.000 PubMed results. This very sensitive method is influenced by a plethora of factors such are patient selection, RNA isolation, chip selection, statistical and bioinformatical analysis as well as independent data validation. To date, a number of various expression profiling studies in patients diagnosed with SpA have been conducted, which were mainly focused on AS patients [11]–[17]. There have also been some studies involving patients with jSpA/ErA [18]–[21]. The results of these studies were often inconsistent, which could be due to patient heterogeneity, different methods of RNA isolation and expression analysis, variations in normalization procedures, poorly preformed bioinformatical analysis and, most of all, a lack of independent data validation. To the best of our knowledge, there has been no expression profiling study with RNA isolated from whole blood in a cohort of patients diagnosed with jSpA using ILAR criteria for ErA, with known HLA genotype and calculated odds ratio for disease development. Furthermore, there has not been a study in which an expression of selected genes was independently confirmed in new cohorts of untreated and treated patients diagnosed with jSpA/ErA, as well as with other forms of JIA. To summarize, the aim of the present study was to identify and confirm gene signatures and novel biomarkers possibly predictive of therapeutic response in various cohorts of untreated and treated patients diagnosed with undifferentiated jSpA and other forms of JIA.

## Materials and Methods

### Patients

Peripheral blood samples were obtained at the Pediatric Rheumatology Clinic in Children's Hospital Srebrnjak from 45 patients with an undifferentiated form of jSpA who fulfilled ILAR criteria for ErA and 11 patients who fulfilled ILAR criteria for oligo- or polyarticular form of JIA [22]. Basic immunological tests (ANA, RF) and HLA genotyping were performed in all the patients diagnosed with jSpA/ErA, as well as spinal MRI in order to exclude the development of axial disease. Twelve gender and age specific blood samples were collected from children with no history of chronic diseases during their visit to the Pediatric Pulmology Clinics at Children's Hospital Srebrnjak.

All the participants were divided in several study groups. In the first group of patients (P1) there were 11 children and in the second (P2) there were 10 children diagnosed with jSpA/ErA whose blood samples were collected 12 to 24 weeks after the development of the first symptoms. These children were treated only with non-steroidal anti-inflammatory drugs (NSAID), with a break in therapy at least 24 hours prior to blood sample collection. In the third patient group (P3) there were 24 children diagnosed with jSpA/ErA whose blood was collected 24 or more weeks after the development of the first symptoms. These patients were treated with standard therapy for jSpA/ErA (NSAID, glucocorticoids, methotrexate). In the fourth group (P4) there was 11 children diagnosed with oligo- or polyarticular form of JIA who were also treated with standard therapy (NSAID, glucocorticoids, methotrexate). None of the patients included in the study received a biological therapy. Participants without a history of chronic disease were divided in two groups depending on the obtained expression profile. In the first group there were four participants (C1) and in the second group there were 12 participants (C2). DNA microarray was performed in the 11 patients diagnosed with jSpA/ErA from P1 group and in the four patients from C1 group. A qRT-PCR confirmation of selected genes was performed in all the participants. Demographic characteristics, diagnosis, performed analysis and HLA genotypes of all the participants are shown in Table 1.

**Table 1:** Demographic characteristics, diagnosis, performed analysis, therapy and HLA genotype of all study participants.

	P1	P2	P3	P4	C1	C2
**Demographics**						
N (m/f)	11 (6/5)	10 (4/6)	24 (11/13)	11 (3/8)	4 (1/3)	12 (6/6)
Age (mean years ±SD)	12.5±3.3	13.6±2.2	13.5±3.8	12.1±4.4	11.7±2.7	13.5±3.3
**Diagnosis**						
ErA/jSpA	11	10	24	0	0	0
Oligoarticular JIA	0	0	0	6	0	0
Polyarticular JIA	0	0	0	5	0	0
**Analysis**						
DNA microarray	11	0	0	0	4	4
qRT-PCR	11	10	24	11	4	12
**Medications**						
NSAID^1^	11	10	24	11	0	0
DMARD^2^	0	0	15	4	0	0
GC^3^	0	0	9	2	0	0
**HLA genotype**						
B7 (OR = 2.61)	1	2	5	NA	NA	NA
B27 (OR = 5.69)	2	6	12	NA	NA	NA
B7/B27 (OR = 14.82)	8	0	1	NA	NA	NA

Odds ratio (OR) for disease development was calculated according to presence of HLA-B27 allele, HLA-B7 allele, or both, HLA-B27 and B7 alleles in a cohort of 74 children from Croatia diagnosed with jSpA/ErA. [7]. It show us that odds for disease development are 2.61 times higher in children with HLA-B7 allele, 5.69 times higher in children with HLA-B27 allele and even 14.82 times higher in children with both HLA-B7 and HLA-B27 alleles than in children from general population without this allele.

1 Non-steroidal anti-inflammatory drugs.

2 Disease-modifying antirheumatic drugs.

3 Glucocorticoids.

### Ethics Statement

The study protocol was in compliance with the Helsinki Declaration and was approved by the University of Zagreb School of Medicine and Children's Hospital Srebrnjak Institutional Review Board (IRB). Parents of all the participants as well as the children older than 12 years have signed an informed consent prior to blood collection.

### RNA isolation

From each study participant 7.5 mL of venous blood was collected using three PAXgene tubes for the stabilization of whole blood RNA (3×2.5 mL = 7.5 mL). Total RNA was isolated from the whole blood using a RNA isolation kit (PaxGene, Preanalytix) at the Department for Functional Genomics, Center for Translational and Clinical Research of the University of Zagreb School of Medicine. All the samples were treated with DNase (RNase-free DNase set, Qiagen, Valencia, CA). The quality of the total RNA was analyzed using RNA 6000 Nano LabChip kit on 2100 Bioanalyser (Agilent Technologies, Santa Clara, CA).

### Gene expression analysis

Microarray analysis was performed using an Affymetrix Human Genome U133 PLUS 2.0 GeneChip (Affymetrix, Santa Clara, CA) according to the manufacturer's protocol. Briefly, 4 µg of high-quality total RNA was reverse-transcribed (Invitrogen, Carlsbad, CA), cleaned with QIAquick purification kit (Qiagen, Valencia, CA), and then used as a template for *in vitro* transcription with T7 MEGA script reagents (Ambion, Austin, TX). The resulting biotin-labeled cRNA was recovered and purified with the RNeasy kit (Qiagen, Valencia, CA), hybridized to the chips, washed, stained on an Affymetrix fluidics station and scanned using an Affymetrix GeneChip scanner. All arrays were run at the Department for Functional Genomics, Center for Translational and Clinical Research of the University of Zagreb School of Medicine.

### Data processing and statistical analysis

After hybridization and scanning of microarray chips, raw signal intensities were obtained from every probe. These raw intensities were subsequently corrected for experimental noise in the signal through the process of normalization. We used MAS5 and GeneChip Robust Multiarray Analysis (GCRMA) normalization procedures and the resulting normalized probe intensities were used to compare differential levels of gene expression between the patient and control samples. Prior to normalization step with GCRMA algorithm, unspecific probes present in Affymetrix microarrays that hybridize to many different mRNA transcripts were removed, and henceforth not considered as a valid detector of the presence of certain mRNA transcript [23]. All the microarray probe intensities processed in this manner were annotated according to Nurtdinov et al., where each probe was labeled according to its specificity and position in the genome [23]. Differentially expressed genes were determined and ranked according to the magnitude of the ratio of expression levels in patient and control samples, with the calculated p-value designating a confidence measure. A correction of the calculated p-value for multiple hypothesis testing was carried out with the Benjamini-Hochberg method for probe intensities normalized with GCRMA algorithm [24]. Finally, a 2-tailed Pearson correlation was performed to compare the expression ratios and p-values of the probes calculated with both of the normalization procedures.

The analysis procedures presented here comply with MIAME guidelines (**M**inimal **I**nformation **A**bout a **M**icroarray **E**xperiment) established by the Microarray Gene Expression Data Society (http://www.mged.org). The data discussed in this manuscript have been deposited in the NCBI's Gene Expression Omnibus (Edgar et al., 2002) and are accessible through the GEO Series accession number GSE58667 ().

### Bioinformatical analysis

A hierarchical clustering was performed using the dChip software on the pool of all samples in order to determine gene clustering and to better visualize differences in expression profiles between the jSpA patients and healthy control subjects. Differentially expressed genes (p<0.05) were functionally annotated and classified with the functional annotation tool of the database for annotation, visualization, and integrated discovery (DAVID) [25], which provides integrated solutions for the annotation and analysis of genome-scale datasets derived from high-throughput technologies.

To determine whether *a priori* defined sets of genes show statistically significant concordant differences between patients and healthy controls, a gene set enrichment analysis (GSEA) using GSEA 1.0 software and >1325 pre-defined sets of genes was performed [26]. The gene sets were defined based on prior biological knowledge, e.g., published information about biochemical pathways or co-expression in previous experiments.

Finally, to associate biological functions and diseases to genes differentially expressed in jSpA/ErA patients the **I**ngenuity **P**athway **A**nalysis (IPA) v.7.6 software was used. The core technology behind this program is the Ingenuity Knowledge Base, a repository of biological interactions and functional annotations created from millions of individually modeled relationships between proteins, genes, complexes, cells, tissues, drugs, and the disease. The IPA “Core Analysis” was performed using the Ingenuity Knowledge Base (Genes only) as the reference set, using direct and indirect relationships for network analysis, and data from mammal species, and all tissues, cell lines and data sources.

### Gene selection for qRT-PCR analysis

Genes for qRT-PCR analysis were primarily selected according to the results of the microarray expression study. Firstly, all genes with the expression ratio >1.5 or <0.66 and p<0.05, as well as genes closely connected with them, were selected for a manual database search (PUBMED/MEDLINE) in order to find studies relevant for those genes. A ranked list of genes potentially important in the pathophysiology of jSpA was then created based on the role of selected genes in chronic inflammatory diseases as well as in various signaling pathways of interest. Genes that were not previously connected with jSpA were ranked higher on the list. Lastly, the results of the enriched functional related gene group's analysis, the gene set enrichment analysis, as well as the pathway and network analysis were accounted for in the definitive gene selection.

### qRT-PCR analysis

qRT-PCR analysis of the ten selected genes was performed for all the participations (the 56 patients diagnosed with jSpA/ErA and other forms of JIA, as well as the 12 healthy controls). The 2 µg of high-quality total RNA extracted from whole blood was reversely transcribed using the SuperScript First-Strand Synthesis system according to the manufacturer's protocol (Invitrogen). The expression of the selected genes was measured with the LightCycler FastStart DNA Master SYBR Green kit (Roche Diagnostics, Mannheim, Germany) and LightCycler instruments (Roche Diagnostics, Mannheim, Germany). The sequences of primers used for the qRT-PCR are shown in Table 2. Relative gene expressions were calculated by the 2^−ΔΔCt^ method, in which Ct indicates cycle threshold, the fractional cycle number where the fluorescent signal reaches detection threshold. The average fold change was computed using the average difference in ΔCt between the genes and internal control, for which β-actin was used.

**Table 2:** Primer sequences used for the qRT-PCR.

Gene	Forward	Reverse
**TLR4**	ATGCTGCCGTTTTATCACGGA	CTAAACTCTGGATGGGGTTTCC
**NLRP3**	GAGGCACAGACAGACCCAGT	AACAGAGCTTGGTGGGTGAG
**PTPRN2**	GCTCCAAAACCAGATGCCTG	GGTCCGAGAACCTCTCTGTCT
**CXCR4**	TGCCCACCATCTACTCCATCA	AGGATGACCAATCCATTGCCC
**TNFSF4**	TGCTCTTCAGGTATCACATCGG	ATGACTGAGTTGTTCTGCACC
**DUSP6**	AGCTCAATCTGTCGATGAACG	GCGTCCTCTCGAAGTCCAG
**MAP2K2**	CCAAGGTCGGCGAACTCAAA	TCTCAAGGTGGATCAGCTTCC
**MAPKBP1**	AAGGGTTTCTCGTCATAATGGC	CGGTTTCCTGCCTTACTCCTG
**MYST3**	TCACCAGCAGTTACGATTGGC	CATCCACGTTGGTTGCTTTAGT
**PTPN12**	CCCAGCGGGAGGTATTCACTATG	GATCTCTTGGTCCTTTGGGTTTTC
**β-ACTIN**	TCCCTGGAGAAGAGCTACGA	AGGAAGGAAGGCTGGAAGAG

## Results

### Global gene expression changes

The Affymetrix platform and statistical analysis with the MAS5 normalization algorithm identified 745 significantly changed genes (target intensity >100, *p*<0.05, average expression ratio >1.5 or <0.66) in 11 jSpA/ErA patients compared with four healthy control subjects. Among these genes, 197 had lower and 548 higher expression. The expression ratio and p-value of probes normalized with MAS5 algorithm showed significant correlation at 0.01 level with the expression ratio of probes normalized with GRCMA algorithm. The correlation coefficient (r) was 0.432 for the expression ratios and 0,249 for the p-value. Among the 745 filtered genes r was 0.854 for the expression ratios and 0.185 for the p-value.

### Gene Ontology analysis using DAVID

The analysis of the enriched functional related gene groups with the DAVID software indicated four gene groups with enrichment score (ES)>1.5 from the list of overexpressed genes and ten gene groups with enrichment score (ES)>3 from the list of underexpressed genes.

In the first group of gene ontology (GO) terms created from the list of overexpressed genes, the majority of the enriched terms were associated to protein kinase activation (CXCR4 gene selected for the qRT-PCR analysis was in this group); in the second group the majority of terms were related to biological processes associated with cell death (CXCR4, TLR4 and NLRP3 genes selected for the qRT-PCR analysis were in this group); in the third group the enriched terms were describing various cell components (DUSP6 gene selected for the qRT-PCR analysis was in this group); finally, in the fourth group the majority of the enriched terms were linked to vacuole, vacuolar membrane and endosome.

In the first group of the GO terms created from the list of the underexpressed genes, the majority of the enriched terms were describing cell components such as various lumens inside cell and cell organelles. The enriched terms in the second group were describing the cell components associated with chromosomes and chromatin. One of the genes in both of these groups was also the MYST3 gene selected for the qRT-PCR analysis. In the third group, the majority of the enriched terms were associated with transcription factors containing Zinc finger and PHD finger motifs. The MYST3 gene was represented in this group as well. In the fourth group the majority of the terms were associated with nucleotide binding (MAP2K2 gene included in the qRT-PCR analysis was in this group). The fifth group of GO terms was chiefly associated with chromatin organization, modification and regulation while the sixth group of genes contained terms related to the biological processes associated with the regulation and control of the cell cycle. In the seventh group the terms were mostly associated with RNA splicing and processing, in the eight group with cell cycle and cell division and in the ninth group the terms were describing DNA damage response, metabolic processes involving DNA and stress associated cell response. Finally, the terms enriched in the tenth group were describing cell components associated with cytoskeleton and organelles not bound to cell membrane. One of the genes in this group was also the MYST3 gene.

### GSEA analysis

The total number of gene sets represented in jSpA/ErA patients identified with GSEA was 634 among which 23 gene sets had a nominal p-value <0.05. The total number of sets with NES>1 was 483. In healthy subjects, the total number of gene sets identified with GSEA was 813, among which 87 gene sets had a nominal p-value <0.05 and 284 sets had NES>1. Only one gene set was significantly enriched with FDR<0.25. Three gene sets were significant in patients with jSpA: the HOX signal pathway, granule the cell survival pathway and the T cell activation pathway. Many genes involved in the HOX signal pathway are also associated with the development of the myelodysplastic syndrome and acute myeloid leukemia, while many genes in the granule cell survival pathway are associated with the MAPK signal pathway. One of the genes involved in the T cell activation pathway set of genes is TNFSF4, which was used for further qRT-PCR analysis.

### IPA network and pathway analysis

Network and pathway analysis with the IPA software showed that the genes differentially expressed in jSpA/ErA patients were involved in multiple functional categories including cell death, gene expression, cellular growth and proliferation, cancer, cellular development, homological system development and function, hematopoiesis, genetic disorder, neurological diseases, DNA replication, recombination and repair, connective tissue disorders and cell mediated immune response. In the primary category of functions related to diseases and disorders, functional categories mostly associated with the genes differentially expressed in jSpA/ErA patients were hereditary disorders as well as inflammatory, infectious and immunological diseases. One of the genes associated with inflammatory and infectious functional categories is also the TLR4 gene, which was selected for further qRT-PCR analysis. In the primary category of processes related to molecular and cellular functions mostly associated functional categories were cell death, gene expression, cell cycle and cellular development. Finally, in the primary category of functions related to the development and functions of the physiological system, functional categories mostly associated with genes differentially expressed in jSpA/ErA patients were hematological system development and function as well as hematopoiesis.

### qRT-PCR analysis

A qRT-PCR data confirmation was performed for six genes that had significantly higher expression in jSpA patients (TLR4, NLRP3, PTPRN2, CXCR4, DUSP6, TNFSF4), for three genes that had significantly lower expression (MAP2K2, MAPKBP1, MYST3) and for one gene that had fold change and p-value very close to the values selected as significant (PTPN12). This gene also has a potentially important but still unclear role in immune diseases. The results of the DNA microarray analysis for the selected genes are shown in Table 3. A qRT-PCR analysis was performed in the same group of patients in which the DNA microarray was performed (P1), in the new groups of treated and untreated jSpA/ErA patients (P2 and P3) as well as in the group of patients diagnosed with oligo- or polyarticular form of JIA (P4), as shown in Table 1. The expression of the selected genes in those groups of patients was compared with the expression in the group of healthy control subject (C2) and the results are shown in Table 4. More specifically, a qRT-PCR analysis of the selected genes in the first group of patients (P1) exhibited a significant (p<0.05) fold change for four genes. Two of them (TLR4, CXCR4) had higher and two (MAPKBP1, PTPN12) had lower expression. In the second group of patients (P2), the qRT-PCR analysis of the selected genes showed significant (p<0.05) fold change for five genes, among which two had higher (TLR4, CXCR4) and three had lower expression (NLRP3, PTPRN2, PTPN12). In the third group of patients (P3), the qRT-PCR analysis showed significant (p<0.05) fold change for five genes. Among these genes one had higher (CXCR4) and four had lower expression (NLRP3, TNFSF4, MAPKBP1, PTPN12). Finally, the qRT-PCR analysis of the selected genes showed no significant fold change in the forth group of patients (P4).

**Table 3:** Results of DNA microarray analysis for genes selected for further qRT-PCR analysis.

Symbol	Name of the gene	Probeset annotation	Intensity measurements	Fold change	p
**TLR4**	Toll-like receptor 4	232068_s_at	248.25	+2.13	0.02
**NLRP3**	NLR family, pyrin domain containing 3	207075_at	130.39	+1.97	<0.01
**PTPRN2**	Protein tyrosine phosphatase, receptor type, N polypeptide 2	203029_s_at	189.93	+1.78	0.01
**CXCR4**	Chemokine (C-X-X motif) receptor 4	217028_at	4753.75	+1.60	0.02
**DUSP6**	Dual specificity phosphatase 6	208892_s_at	1724.53	+1.71	0.05
**TNFSF4**	Tumor necrosis factor (ligand) superfamily, member 4	207426_s_at	137.50	+1.95	<0.01
**MAP2K2**	Mitogen-activated protein kinase kinase 2	213490_s_at	193.45	−1.55	0.03
**MAPKBP1**	Mitogen-activated protein kinase binding protein 1	213394_at	130.88	−2.17	0.01
**MYST3**	MYST histone acetyltransferase (monocytic leukemia) 3	216361_s_at	101.78	−3.26	<0.01
**PTPN12**	Protein tyrosine phosphatase, non-receptor type 12	202006_at	710.88	+1.57	0.07
		216884_at	6.09	−1.05	0.91
		216915_s_at	26.69	+2.30	0.11

Every gene on the GeneChip is represented with one or more probe set. The intensity of every probe set can be measured and the ratio of probe set intensities on experiment and baseline arrays is used for fold change calculation. In order to show significant differences in probe set intensities, the p-value is calculated. Presented fold change and p-value were calculated after probe set intensities normalization with MAS5 algorithm.

**Table 4:** Results of qRT-PCR analysis for selected genes in all group of patients.

Gene	P1		P2		P3		P4	
	FC	p	FC	P	FC	P	FC	p
**TLR4**	**+4,36**	**0,05**	**+3,91**	**<0,01**	+1,32	0,26	+1,37	0,30
**NLRP3**	+2,06	0,53	**−5,00**	**<0,01**	**−3,70**	**0,05**	−2,13	0,21
**PTPRN2**	+2,60	0,54	−5,55	0,01	+1,41	0,59	−2,78	0,17
**CXCR4**	**+1,89**	**<0,01**	**+1,87**	**<0,01**	**+1,37**	**0,03**	+1,23	0,43
**DUSP6**	−1,12	0,72	−1,19	0,52	+1,11	0,66	−1,06	0,71
**TNFSF4**	+1,02	0,97	−1,16	0,46	**−1,56**	**0,04**	+1,26	0,23
**MAP2K2**	−1,25	0,44	1,04	0,80	−1,07	0,75	+1,01	0,96
**MAPKBP1**	**−3,12**	**0,02**	−1,39	0,29	**−1,96**	**0,05**	−1,05	0,75
**MYST3**	−1,10	0,73	−1,05	0,79	+1,03	0,86	−1,56	0,34
**PTPN12**	**−3,70**	**<0,01**	**−2,63**	**<0,01**	**−2,44**	**<0,01**	−1,06	0,84

Fold change (FC) represents the relative increase (+) or decrease (−) of expression calculated using the ^−ΔΔCt^ method, and p stands for the calculated p-value.

## Discussion

The present study differs from previous studies in a variety of methodological ways such as the avoidance of pooling all JIA subtype patients together, the use of whole blood (which avoids the potential artifact induced by isolating leukocytes or leukocyte subsets), and a much larger number of the transcripts studied per patient (54.675 probe sets). To our best knowledge this is the first study to comprehensively evaluate whole blood gene expression patterns in undifferentiated jSpA/ErA patients diagnosed with ILAR classification criteria for JIA. All of the patients were younger than 16 years of age at the time of the disease onset and they also fulfilled ESSG and Amor criteria for SpA. Patients with AS, PsA, ReA and IBD were excluded while the aim of the present study was to show the activity of the genes in undifferentiated patients which still can progress or regress to any of the mentioned forms of SpA. This allowed us to compare the gene activity in differentiated and undifferentiated patients; e.g. TLR4 and CXCR4 were overexpressed in the present study, as well as in other gene expression studies performed in patients diagnosed with AS and PsA [11], [16]. The full impact of this could come after the gene expression profiling is repeated in the same cohort of patients over the course of time, which we plan to do. It is unfortunate that there was no statistically significant difference in the expression of genes whose polymorphisms are associated with the risk for development of AS [8]. One of the possible explanations is that SNPs has to be in special loci inside the gene, the so-called expression quantitative trait loci (eQTL), to influence gene expression [27]. Further on, gene expression may not be the only mediator for the relation between SNPs and the disease, while other biomarkers, such as DNA methylation, proteins, metabolites of the gene product in the blood, immunological or biochemical markers in the serum, and environmental factors can also serve as potential mediators, depending on the context or the disease to be studied [28].

### Spondyloarthritis and HLA genes

The dominance of a single gene in a complex genetic disease places HLA-B27 and spondyloarthritis in an unusual place among immune-mediated and autoimmune inflammatory disorders [3]. Previous studies have shown that ERAP and HLA-B27, but not HLA-B7, jointly contribute to predisposition for AS in animal model, and that HLA-B7 is associated with undifferentiated SpA in the HLA-B27 negative group of patients [29], [30]. The majority of jSpA patients participating in our study had the HLA-B27 and/or HLA-B7 alleles with significant OR for disease development, which was the highest when the both antigens were present (see introduction). However, as already explained, the HLA genes are only a few of the many genes involved in the disease pathogenesis and our study pointed to other important contributors, many of which were never before put in the context of jSpA/ErA.

### Bioinformatical analysis

To elucidate genes involved in this complex disease we preformed a whole transcriptome analysis. Due to the vast amount of data gathered in such a manner it is very important to select an appropriate analysis that could lead us to valuable conclusions. Thus we performed several different bioinformatical analyses that gave some biological meaning to the results of the microarray experiment. The analysis of the enriched functional related gene groups showed that the blood cells of patients with jSpA/ErA, from which RNA was isolated, have higher activity of various processes connected with apoptosis and lower activity of processes connected with transcription and cell cycle, which could indicate dysfunction of this cells on many levels. It is important to point out that six genes (CXCR4, TLR4, NLRP3, DUSP6, MYST3, MAP2K2) were selected for further validation based on the results of this analysis. GSEA showed that many genes differentially expressed in the blood cells of study patients are connected to processes believed to be important in pathogenesis of SpA, such as gene expression, morphogenesis, cell differentiation, Wnt and MAPK signal pathway and T cell activation. Based on the results of a GSEA analysis we selected the TNFSF4 gene for further validation. Finally, the results of network and pathway analysis lead us to conclusion that mechanisms similar to those observed in inflammatory, genetic, immunological and infectious diseases also have important role in development of jSpA/ErA. The results of this analysis once more underlined the importance of TLR4 gene.

### Statistical analysis

When evaluating microarray expression data, we must ask whether the results are accurate for the particular biological system under study and do the data fundamentally describe the phenomenon being investigated [31]. Since there is still no consensus about the most appropriate normalization procedure for microarray experiments, we used the two most common algorithms. Unfortunately, correlation coefficients of expression ratios and especially of p-values calculated after normalization procedures with both algorithms were not especially high.

### qRT-PCR analysis

To independently confirm data gathered in the microarray experiments, ten genes were carefully selected for a qRT-PCR analysis in the same group of patients in which microarray was preformed. Further on, for confirmation of data universality the same genes were analyzed in new groups of untreated and treated patients diagnosed with jSpA/ErA. Finally, to confirm that the retrieved data were specific for patients with jSpA/ErA, the qRT-PCR analysis of the selected genes was performed in a group of patients diagnosed with other forms of JIA.

### Role of the genes selected for further analysis

TLR-4 mediates NF- κB activation, cytokine secretion and the inflammatory response [32]. Serum TLR-4 protein and mRNA levels, as well as TLR-4 gene expression were previously found to be significantly higher in adult AS patients [16], [33]. In our study, this gene was overexpressed only in cohort of untreated (P1 and P2), but not in treated (P3) patients, which could mean that the standard therapy or the course of the disease influence the expression of this gene.

Product of CXCR4 is a chemokine receptor with a previously described role in the pathophysiology of SpA [34]. CXCR4 and its ligand CXCL12 have been suggested to be important in retaining inflammatory cells within the joint, and recently have been incorporated into the pathogenesis of IBD [35], [36]. Since it was overexpressed in all of our jSpA/ErA patients (P1–P3) we believe it might contribute to the mucosal disregulation, leading to “low-grade IBD”. This is in accordance with the hypothesis that the gut is the primary site of pathology and that the recirculation of the activated immune cells from the gut to musculoskeletal sites leads to entheseal complex and joint inflammation [37]–[39].

The reduced expression of NLRP3 gene is a novel observation in jSpA/ErA and is especially intriguing. Polymorphisms in other NLR and related genes have been implicated in diseases that share clinical features with SpA, including Behçet's disease, Crohn's disease and psoriatic arthritis, while reduced NLRP2 expression was found in adults with axial spondyloarthritis [13]. Recent studies suggest that a defective NLRP3 inflammasome signaling in the gut contributes to IBD through increased permeability across the epithelial barrier and the induction of detrimental immune responses against invading commensals [40]. Therefore, the downregulated NLRP3 gene in jSpA/ErA patients might reflect development of a subclinical inflammation of the gut mucosa (“low-grade IBD”), leading to a leaky gut.

Three different PTPs were selected for further qRT-PCR analysis in our study (PTPRN2, DUSP6, PTPN12), and we found lower expression of PTPN12 in all cohorts of jSpA/ErA patients. According to previous studies, PTPN12 is a negative regulator of inflammation and intestinal cell migration, as well as a positive regulator of osteoclast activity, all of which are processes important for the patophisiology of jSpA/ErA [41]–[44]. A recent oncological study showed that the PTPN12 gene could be silenced by methylation, giving a possible explanation for the PTPN12 downregulation [42].

Other genes selected for the qRT-PCR analysis could potentially also have important role in the jSpA development. MAP2K2 and MAPKBP1, along with majority of the other selected genes, are involved in NFκB and MAPK signaling pathways, known to be important for the regulation of the immune response [45]. TNFSF4 is one of the costimulatory molecules important for the lymphocyte activation, while MYST3 is involved in epigenetic regulation [46], [47].

It is important to stress that none of the genes showed a statistically significant expression difference in other JIA patients (P4), possibly meaning that the observed expression profiles are specific for jSpA/ErA patients.

### Important limitations

The weakness of microarrays is its susceptibility to great experimental variability and the lack of a single unanimously accepted optimal method of statistical analysis. An important consideration in the interpretation of gene expression profiles obtained from complex mixtures of cells is the influence of cellular composition. Genes that are referred to as ‘up- or down-regulated’ may actually be ‘over- or under-represented’ due to differences in abundance of cell populations [20]. High-throughput technologies are by their nature hypothesis generating and the hypotheses presented in this study must be validated in other cohorts while the biological relevance of gene expression differences identified here remain to be determined at protein and organism levels. Nevertheless, the results of the microarray analysis in the present study were validated by qRT-PCR, which is referred to as a golden standard method for gene expression measurement [48]. Validation was performed in the new cohort of treated and untreated patients diagnosed with jSpA, as well as in the cohort of patients diagnosed with other forms of JIA. This independent confirmation of results, as well as confirmation of data universality and specificity for jSpA patients, gave more significance to microarray analysis results [31]. Unfortunately, the microarray expression was not confirmed by the qRT-PCR in jSpA/ErA patients group for MYST3, DUSP6 and MAP2K2, while it was not consistent for NLRP3, PTPRN2, TNFSF4 and MAPKBP1. Interestingly, the PTPN12 gene showed a statistically significant expression in all groups of jSpA/ErA patients when analysis was preformed with qRT-PCR, but not with microarray. We believe this is possibly due to a small number of patients, which is another important limitation of the study. Finally, a major limitation of our study is a lack of evidence of importance for the identified markers with *in vitro* assays or *in vivo* animal studies.

### Conclusions

The present study has identified differences in the expression of genes related to various processes responsible for jSpA/ErA development. Among these, the genes of highest importance were TLR-4 and NLRP3, which can lead to the development of systemic autoimmunity, as well as CXCR4 and PTPN12, which can drive other processes important for the pathophysiology of jSpA/ErA. Several differentially expressed genes (e.g. TLR-4, CXCR4) replicate findings in other studies providing further confidence in ours [11], [16], [49]. We strongly believe that our results represent an important step toward a better understanding of the molecular mechanisms and complex pathophysiology of jSpA/ErA. The highlighted genes and their products could have a prognostic value and potential therapeutic use, although further research at the level of epigenome and proteome in a larger cohort of patients are needed to make this feasible.
